# Stochastic model predicts evolving preferences in the Iowa gambling task

**DOI:** 10.3389/fncom.2014.00167

**Published:** 2014-12-19

**Authors:** Miguel A. Fuentes, Claudio Lavín, L. Sebastián Contreras-Huerta, Hernan Miguel, Eduardo Rosales Jubal

**Affiliations:** ^1^Santa Fe InstituteSanta Fe, NM, USA; ^2^Instituto de Sistemas Complejos de ValparaísoValparaíso, Chile; ^3^Instituto de Investigaciones Filosóficas and CONICET, Sociedad Argentina de Análisis Filosófico (SADAF)Buenos Aires, Argentina; ^4^Facultad de Economía y Empresa, Centro de Neuroeconomía, Universidad Diego PortalesSantiago, Chile; ^5^Faculty of Psychology, Centre for the Study of Argumentation and Reasoning, Universidad Diego PortalesSantiago, Chile; ^6^Laboratory of Cognitive and Social Neuroscience (LaNCyS), UDP-INECO Foundation Core on Neuroscience, Universidad Diego PortalesSantiago, Chile; ^7^Universidad de Buenos Aires, Sociedad Argentina de Análisis Filosófico (SADAF)Buenos Aires, Argentina; ^8^Department of Neurophysiology, Max Planck Institute for Brain ResearchFrankfurt am Main, Germany; ^9^Ernst-Strüngmann Institute for Neuroscience in Cooperation with the Max Planck SocietyFrankfurt am Main, Germany; ^10^Focus Program Translational Neurosciences, Institute for Microscopic Anatomy and Neurobiology, Johannes Gutenberg-University MainzMainz, Germany

**Keywords:** Iowa gambling task, uncertainty, decision making, stochastic, learning, categorization, dynamic landscape, conceptual network

## Abstract

Learning under uncertainty is a common task that people face in their daily life. This process relies on the cognitive ability to adjust behavior to environmental demands. Although the biological underpinnings of those cognitive processes have been extensively studied, there has been little work in formal models seeking to capture the fundamental dynamic of learning under uncertainty. In the present work, we aimed to understand the basic cognitive mechanisms of outcome processing involved in decisions under uncertainty and to evaluate the relevance of previous experiences in enhancing learning processes within such uncertain context. We propose a formal model that emulates the behavior of people playing a well established paradigm (Iowa Gambling Task - IGT) and compare its outcome with a behavioral experiment. We further explored whether it was possible to emulate maladaptive behavior observed in clinical samples by modifying the model parameter which controls the update of expected outcomes distributions. Results showed that the performance of the model resembles the observed participant performance as well as IGT performance by healthy subjects described in the literature. Interestingly, the model converges faster than some subjects on the decks with higher net expected outcome. Furthermore, the modified version of the model replicated the trend observed in clinical samples performing the task. We argue that the basic cognitive component underlying learning under uncertainty can be represented as a differential equation that considers the outcomes of previous decisions for guiding the agent to an adaptive strategy.

## 1. Introduction

Uncertainty is related to the probabilities of receiving outcomes and the evaluation of the obtained results (Hsu et al., 2005). Whereas the probabilities of getting heads and tails when tossing a coin are known, the actual result of a specific trial is highly uncertain (Jaynes and Bretthorst, 2003). Furthermore, we are often presented with different options for which we have no cues or associations regarding their true probabilities. Within such contexts, learning by experience is a crucial process for adapting our behavior and performing decisions successfully (Rangel et al., 2008; Gluth et al., 2013).

A widely used experimental task for observing decision making under uncertainty in both healthy and clinical populations is the Iowa Gambling Task (IGT) (Bechara et al., 1994). The IGT is an experimental paradigm that seeks to emulate real-life decision making under uncertainty (Bechara et al., 1994). In this task, subjects have to choose one card at a time from one of four decks presented (decks A, B, C, and D), knowing only that they will gain money with every selection, and that sometimes a loss could follow the gain. They are instructed to maximize their winnings. Participants have to learn the magnitudes of the winning as well as the probabilities and magnitudes associated to the losses by trial and error. Unknown to participants, two of the four decks are more rewarding in the long term (C and D), presenting lower magnitude of winnings but also lower losses, resulting in higher net winnings.

Successful performance on the IGT is associated with functional outcome–monitoring processes, error detection, executive functions and the usage of previous information for future decisions (Hooper et al., 2004; Turnbull et al., 2005; Weller et al., 2009). The learning mechanism of the IGT requires the activity of a complex decision making circuitry, which includes the dorsolateral prefrontal cortex (dlPFC), the insula, anterior and posterior cingulate cortex (ACC and PCC respectively), orbitofrontal (OFC) and ventromedial prefrontal cortex (vmPFC), striatum, and amygdala (Li et al., 2010). The cognitive activity of this circuitry involves working memory processes, the representation of emotional states about the outcomes (particularly in the case of the punishments), and action planning derived from the monitoring processes (Martinez-Selva et al., 2006; Li et al., 2010). Thus, people's ability to adapt their behavior within uncertain contexts is thought to rely on the normal functioning of the different components of this network.

The Somatic Marker Hypothesis (SMH) provides a cognitive framework for understanding how the aforementioned structures and functions work in situations of high uncertainty, allowing people to perform according to long-term goals (Bechara et al., 1994, 1996; Martinez-Selva et al., 2006). The SMH proposes that somatic states triggered by emotional responses are crucial for understanding the underlying cognitive processes involved in learning within uncertain environments (e.g., outcome feedback). These somatic responses occurring after a given outcome feedback will act as anticipatory responses in order to avoid unwanted options based upon previous experiences (Bechara et al., 1994, 1996; Martinez-Selva et al., 2006). Whereas the amygdala is the responsible for these automatic emotional responses triggered by feedback information, prefrontal structures play an important role in triggering somatic states evoked by memories and knowledge in order to adapt behavior toward beneficial strategies (e.g., remembering that a given strategy was ineffective in the past) (Bechara, 2005). Thus, the basic processes of decision making under uncertainty require emotional responses triggered by feedback (amydgala activity) and high-level cognitive processes such as working memory (prefrontal activity) that bring online knowledge for the deliberation of decisions.

Impaired behavior in the IGT has been related to a lack of emotional anticipatory responses associated with risky decisions and is congruent with subjects' impaired adaptive behavior in other contexts, likely due to damage to vmPFC/OFC areas. Unlike healthy participants, subjects with damage in vmPFC/OFC areas performing the IGT choose significantly more from the disadvantageous decks than the advantageous ones. This impairment is believed to be specific to those areas because patients with prefrontal lesions, which differ from vmPFC/OFC lesions, perform well in the task (Bechara et al., 1998; Anderson et al., 1999; Bechara and Damasio, 2002; Bechara, 2003, 2004; Bechara and Martin, 2004; Fellows, 2004). vmPFC/OFC patients may not display anticipatory somatic responses while performing the task and therefore do not receive psycho-somatic feedback about their risky decisions, making them more susceptible to being lured by the higher reward magnitudes associated with the disadvantageous decks (Bechara et al., 1996). In fact, such anticipatory responses have been correlated with good performance in similar gambling tasks (Carter and Smith Pasqualini, 2004; Crone et al., 2004), supporting this interpretation. Therefore, the evidence suggests that subjects with damage in the vmPFC/OFC areas are insensitive to future consequences of their decisions (Bechara et al., 2000a,b).

Within the context of decision making under uncertainty the function of both vmPFC and OFC is also associated with controlling impulsive and automatic behavior (Berlin et al., 2004; Bechara, 2005; Boes et al., 2009). In the IGT, participants must learn over time to inhibit the temptation of higher immediate rewards (certain result) by learning to avoid higher punishments (uncertain result). Thus, the impairment of cognitive functions related to the integration of previous experiences may lead subjects to prioritize immediate feedback rather than longer-term profitability (Rescorla, 1997). Previous studies (Rescorla, 1997; Berlin et al., 2004; Bechara, 2005; Martinez-Selva et al., 2006; Boes et al., 2009) showed the importance of the integrating previous emotional experiences and controlling impulsive responses in the learning process within uncertain environments. In line with this previous work, psychiatric populations with symptoms of impulsivity and anxiety exhibit impaired performance on the IGT. For example, patients with substance use disorders have shown significantly lower performances on the IGT compared with healthy participants (for a review see Buelow and Suhr, 2009). Interestingly, these findings are converging with animal studies using adapted versions of the IGT (Pais-Vieira et al., 2007; de Visser et al., 2011). Rats with lesions in the OFC showed persistence in choices associated with higher magnitude but low probability rewards, which authors have interpreted as indicative of disrupted risk assessment (Pais-Vieira et al., 2007).

In the present work, we aim to understand the basic cognitive mechanisms of outcome processing involved in decisions under uncertainty by evaluating the relevance of previous experiences in enhancing learning processes. In order to do this, we propose a dynamical model that emulates the features of learning-by-experience processes that we compare with IGT performance in a sample of healthy subjects. There are antecedents of models using different parameters in order to emulate normal and biased behavior in the IGT. For instance, the Expectancy Valence Model (EV) (Busemeyer and Stout, 2002) modulates IGT performances through three parameters related to the weighting of losses and rewards, the influence of past experiences and a consistency parameter which determines the amount of exploration vs. exploitation (Steingroever et al., 2013). A recent study of this model modulating its parameters within a given range of variability showed that its main choice pattern is the selection of convenient decks over the bad ones, but that in the second place the choice pattern present in the model is the election of bad decks over good ones (Steingroever et al., 2013). The Prospect Valence Learning Model (PVL) (Ahn et al., 2008) is another model that includes the prospect utility function from prospect theory (Tversky and Kahneman, 1992) in two parameters. The parameter *A* determines the shape of the utility function and parameter *w* determines the loss aversion. Thus, if *A* is close to 0 the utility function can approaches a step function which involves that given a positive or negative net outcome all utilities become similar, but if *A* approaches 1 then the subjective utility increases/decreases in proportion to the net outcome (Steingroever et al., 2013). Another difference between this model and the EV is that in this model on every trial the utilities of every deck are updated. The modulation of this model's parameters has shown that its main choice patter is the election of good vs. bad decks in the IGT (the same than model EV), and also the election of infrequent-losses decks (B y D) over decks with frequent losses (A y C). There is combination of these models (EV-PU model) which uses the utility function of the PVL model, but maintains the rest of the parameters of the EV model. The EV-PU shows a similar pattern of choices than the PVL model (Steingroever et al., 2013).

The model that we propose is a discrete version of a stochastic differential equation (Fuentes and Miguel, 2013), which considers the probability of success given previous outcomes, the stochastic nature of the task, and errors on information computation. This model updates the weight of each deck as a function of the positive minus negative feedback (the deck net value), and then all probabilities are normalized using the new values based on the previous results (see Equation 2 below). Thus, the model weights equally rewards and losses, in a simpler way than the EV and PVL model do. However, given the normalization of each deck probabilities based upon the new value of the chosen deck, then all probabilities are updated as in the PLV model (see Equation 3, below). Our model includes also a parameter that determines the rate of exploration (e), if it gets close to zero then there is absence of exploration (and the first choices will determine the entire decision process), and if gets close to 1 then the choice pattern is random. For the simulations that we ran this parameter value was 0.2, and similar results are found using similar values.

Additionally, we modulate the parameter responsible for outcome processing to create a second condition where we contrast whether this manipulation is sufficient to emulate a maladaptive bias behavior that has been observed in clinical populations. This modification is based upon the evidence that relates impairment performances in the IGT to a lack of impulsive behavior control and poor integration of negative past experiences (Bechara et al., 1994; Turnbull et al., 2005; Li et al., 2010). By doing so, we can observe, firstly, the basic computational mechanisms of learning within uncertain environment, and secondly, the accuracy of the model for simulating decision-making under uncertainty.

We hypothesize that the cognitive dynamics governing strategy deployment in the IGT can be captured by a parsimonious model including a parameter representing deck-choosing-probability that is updated on a trial-by-trial basis with positive and negative outcome information. If this assumption stands, we predict that the model will emulate the behavior deployed by samples of healthy participants completing the IGT. Specifically, the model will resemble the behavioral dataset of the IGT. Conversely, the model version based on the equation that guides decisions using just information of positive but not negative feedback will misguide the agent toward an inconvenient strategy resembling the behavior described in clinical populations.

## 2. Stochastic model

The formal model proposed here considers a discrete dynamics inspired by a recently introduced stochastic dynamics (Fuentes and Miguel, 2013).

In order to take into account the four different deck options, or positions: *k* = *A*, *B*, *C*, *D*, of the subject completing the IGT, we will propose a variation of the previous mentioned model, suitable for the present case. The dynamics of the model can be arranged in the following way: given the deck position at time *t*, the chosen deck at time *t* + 1, say the deck *k*, will be the one that satisfy the following equation

(1) $$k\;/\;max[P_{k}(t)-P_{i}(t)+\xi_{k}(t)]\;\forall \;i=A,B,C,D.$$

meaning that the chosen deck at time *t* + 1 will be the one that maximizes this stochastic gradient-type dynamic for the probability. Notice that the same gradient dynamic was present in the previous mentioned continuous case (Fuentes and Miguel, 2013). Here ξ_*k*_(*t*) is a Gaussian white noise of zero mean and delta correlated, i.e., < ξ_*k*_(*t*) ξ_*k*_(*t*′) > = ε δ(*t* − *t*′), ε is the intensity of the noise, that models the agent's uncertainty at each election (we have used ε = 0.2 in all simulations). *P*_*k*_(*t*) and *P*_*i*_(*t*) are the probabilities of being in the decks *k* or *i* (calculated from the visiting frequency, i.e., the number of times each deck was chosen).

Because at each time only one of the four possibilities is selected, the associated probability evolves using these occupancies. If the agent receives at each time a positive feedback along with a possible negative feedback, denoted by α(*t*), then the probability at that position will increase or diminish proportionally to that information. In the case of a normal agent, α(*t*) will be equal to the sum of the gain and the punishment, (g, l) in Table 1 below, while in the case of maladaptive behavior only the gain will be considered. In general, this is represented by

(2) $$P_{i}(t+1)\;=\;\frac{c_{i}(t)}{\sum_{j\;=\;1}^{4}c_{j}(t)}\;,$$

where *c*_*i*_ is the aggregate of all the times the position *i* was visited

(3) $$c_{i}(t)\;=\;\sum_{t\;=\;1}\alpha (t)\;\text{if}\;c_{i}(t)\geq 0\;\text{; or }c_{i}(t)=0\;\text{if}\;\sum_{t\;=\;1}\alpha (t)<0\;.\;$$

**Table 1:** **Distribution of positive (gain, *g*) and negative (punishment, *l*) feedback in the IGT**.

**Deck**	**(***g***_**1**_, ***l***_**1**_)**	**(***g***_**2**_, ***l***_**2**_)**	**(***g***_**3**_, ***l***_**3**_)**	**(***g***_**4**_, ***l***_**4**_)**	**(***g***_**5**_, ***l***_**5**_)**	**(***g***_**6**_, ***l***_**6**_)**	**(***g***_**7**_, ***l***_**7**_)**	**(***g***_**8**_, ***l***_**8**_)**	**(***g***_**9**_, **l**_**9**_)**	**(***g***_**10**_, ***l***_**10**_)**
A	(1, 0)	(1, 0)	(1, 0)	(1, 0)	(1, −12.5)	(1, 0)	(1, 0)	(1, 0)	(1, 0)	(1, 0)
B	(1, 0)	(1, −2.5)	(1, 0)	(1, −2.5)	(1, 0)	(1, −2.5)	(1, 0)	(1, −2.5)	(1, 0)	(1, −2.5)
C	(0.5, 0)	(0.5, −0.5)	(0.5, 0)	(0.5, −0.5)	(0.5, 0)	(0.5, −0.5)	(0.5, 0)	(0.5, −0.5)	(0.5, 0)	(0.5, −0.5)
D	(0.5, 0)	(0.5, 0)	(0.5, 0)	(0.5, 0)	(0.5, −2.5)	(0.5, 0)	(0.5, 0)	(0.5, 0)	(0.5, 0)	(0.5, 0)

*Note that distributions of reward and punishment are identical as in the original task, where net values per 10 cards in decks A and B is –2.5, whereas for deck C and D is 2.5*.

Therefore, if at time *t* the deck *i* was chosen then *c*_*i*_(*t*) will be the value solely associated with the gain or with the value associated with the gain minus the loss; obviously, if at time *t* the deck *i* was not chosen *c*_*i*_(*t*) = 0. The condition (3) shows clearly, by definition, that *c*_*i*_(*t*) ≥ 0.

Then, the probabilities will be updated as showed in Equations (2) and (3). Note that α(*t*) accounts for the positive of negative feedback obtained when certain deck is chosen and constitutes the substantial factor for updating the probability assigned to that deck after choosing it. So, we can manage that factor to adjust different ways of changing the updating for different individuals. If α(*t*) takes into account both gains and losses (considering equally gain and losses), the resulting update models the normal expected behavior for an individual, and if takes into account only the gains, or the gains weights more than the losses, we expect the model will emulate a choice pattern observed in pathological behavior (maladaptive condition). Therefore, α(*t*) is the input for obtaining *c*_*i*_(*t*), the sum of all historical feedbacks gained for the deck along the whole trial. Then, the way that α(*t*) contribute to *c*_*i*_(*t*) makes de difference, being α(*t*) the parameter to be adjusted for modeling different kind of behavior.

## 3. Materials and methods

This study consists in a behavioral experiment of the IGT in a sample of normal subjects and a computational simulation consisting of three conditions. First, we tested the stochastic model on the IGT version used in the behavioral experiment (Lavin et al., 2014). Secondly, we modified the model while keeping the same IGT condition to simulate biased decision-making patterns observed in clinical (Anderson et al., 1999; Bechara and Damasio, 2002; Bechara, 2003, 2004; Bechara and Martin, 2004; Fellows, 2004) and psychiatric samples (Buelow and Suhr, 2009). Third, we ran a control simulation on a modified version of the IGT, where the distribution of positive and negative feedback were equal for the four decks.

### 3.1. Experimental task

We used a modified version of the IGT for both the simulation and the behavioral experiments. The underlying distribution of rewards and punishments was maintained from the original IGT (Bechara et al., 1994), with small changes in the monetary quantities of the feedback. The task consists of an iterated card selection from four virtual decks (named A, B, C, and D respectively) with different distributions of winnings and losses. Decks A and B always presented a reward of $100. In addition, deck A had a punishment of $250 with 0.5 probability, B had a punishment $1250 with 0.1 probability. Both decks had a net value (NV) of $250 per 10 card selections. The modification of the traditional version of the IGT involved the standardization of deck A's punishment to $250, whereas in the original version it fluctuated between $150 and $350. This change did not affect the NV, which remained the same. This was done in order to exclude possible effects produced by the novelty of having different feedback magnitudes in each trial of the same deck. Decks C and D always presented a reward of $50. Whereas deck C had cards with a punishment of $50 with a 0.5 probability, the D deck cards punished with $250 with 0.1 probability. Both decks had a net value of $250, being the advantageous options for participants. The values of punishing cards in deck C were changed in the same way than the deck A and for the same reasons. Again the NV was maintained as in the original IGT.

### 3.2. Simulation

As described above, the simulation was run for three different conditions. The first condition replicated the task used in the normal group of subjects. We tested the model using the same version of the IGT as in the behavioral experiment. Thus, the model sought to emulate the scenario that our sample faced in order to compare the learning-by-experience process observed in healthy subjects. In the second condition we modified the outcome processing of the model in order to bias its decision-making. The modification consisted of the processing of each desk's payoffs to be based on a constant punishment value. This modification was done in order to emulate the cognitive damage observed in patients with absence of anticipatory somatic responses. This damage, associated with the vmPFC, is thought to represent the neural correlates for the inability of updating loss probability in order to avoid risky decisions (Bechara et al., 1994; Martinez-Selva et al., 2006). Given that the inconvenient decks present higher winnings but also higher losses, these patients are unable to use contextual information in order to adapt their behavior. Thus, we sought to determine whether the simulation replicates the behavior of patients who are unable to learn from task contingencies.

The third condition measured the model's performance in a version of IGT with identical payoffs for all decks. This control condition replicated the context of the IGT (a series of selections over four decks), but included an equal distribution of positive and negative feedback across the four decks. This modification was made in order to observe the model's distribution of choices within a decision-making context where there were no incentives to bias the decision toward any of the decks. In other words, there was nothing to learn for the model. This control condition was thought to verify that the final card selection pattern arose as an interaction between the selection strategy of the system, in this case, the model, and the underlying distribution of rewards and punishments present in the four decks. This underlying distribution represents the environment where the system is exerting its choices and represents the source of feedback for such choices.

All simulations were performed using Wolfram Mathematica 8.0 and its functions (e.g., random functions). Each deck of cards was arranged in a series of ten cards. Each condition of the model ran for 200 trials and 10 iterations, representing 10 subjects playing an IGT version with 200 trials.

### 3.3. Behavioral experiment

The experimental procedure is reported in Lavin et al. (2014). 10 healthy participants (5 women) performed an adaptation of the IGT (Bechara et al., 1994). Similar to the original IGT, subjects started with a virtual loan of $2000. They were told that the game consisted in a long set of card selections, one card at a time. Subjects were asked to pick one card from four virtual decks (A, B, C, and D). They were instructed that cards from each deck always involved a standard winning amount (following card selection and varying across decks) and that sometimes they may receive a punishment (appearing after the positive feedback, and varying across decks) mixed in with the winning cards. Participants had 5 s to select a card from one of the four decks. If they did not choose any card then the trial was repeated. After selection of the card, positive feedback was presented for 3 s and occasionally followed by negative feedback presentation for 3 s.

Participants played two series of 100 trials each with a 5 min break between them. The instructions were preserved from the original IGT, where participants were told that the goal of the game was to maximize their winnings, and they were free to switch from one deck to another at any time. Subjects were not told about the probability and magnitude distribution over the decks or how many trials they had to play. They were told that there was a base payment of €15 for their participation, followed by a bonus based on their performance. However, all the subjects were equally rewarded with €25 regardless of their performance at the end of the experiment. Information about the payoffs was provided to the subjects after the experiment.

## 4. Results

### 4.1. Behavioral experiment

**Figure 1:**
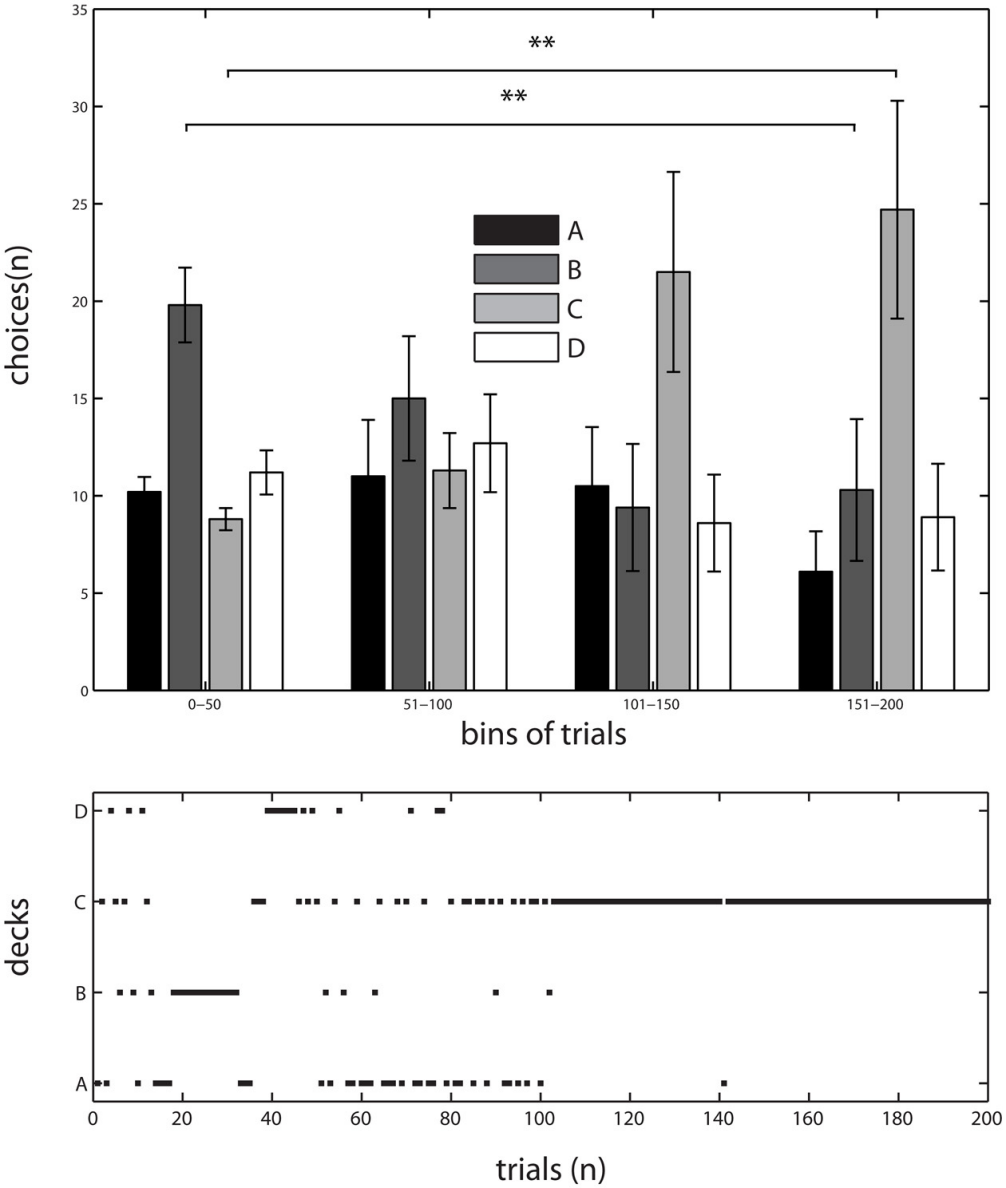
**Behavioral results of a version of IGT**. **Top**, Choices probabilities of the participants across the experiment, pooled in bins of 50 trials. Data as Mean ± S.E.M. ^**^: significant differences in *post-hoc t*-test; deck B was chosen significantly more by subjects in the first 50 trials than in the last 50 selections (paired *t*-test (9) = 3.1540, *p* < 0.01). Whereas in deck C an inverse relation was found, subjects chose it more frequently toward the end of the task compared to the first bin (paired *t*-test (9) = 2.9198, *p* < 0.01). **Bottom**, Evolution of choices through the experiment in a typical subject, note the normal bias following general trend observed in A. Adapted from Lavin et al. (2014).

Results of the behavioral experiment were reported in Lavin et al. (2014). The temporal evolution of subjects' choices were pooled in 50 trials' bins (which we call blocks) out of the total of 200 trails. Briefly, we submitted the data to a Two-Way ANOVA, with time (blocks) and deck as factors. We found a main effect of deck [*F*_(3, 144)_ = 4.73, *p* < 0.05] as well as a significant interaction between time and deck [*F*_(9, 144)_ = 3.4, *p* < 0.001], which was interpreted as a sign of learning because participants' choices depended on how long they were performing the task. The interaction between time (blocks) and deck selection shows that deck C was progressively the most selected by subjects, whereas deck B started as the most popular but selection from this deck decreased toward the end of the task (see Figure 1). Since the IGT structure comprises two advantageous decks (C and D) and two disadvantageous ones (A and B), task learning is expressed when subjects increasingly select from the advantageous decks over the disadvantageous ones as the task progresses (Bechara et al., 1994). Subjects' preference toward deck C suggests that participants indeed pursued a strategy to maximize their benefits from the task. We confirmed this observation by performing *post-hoc* paired *t*-test between the frequency of selections in the first and last bins of the task for decks B and C. We found that deck B was chosen significantly more by subjects in the first 50 trials than in the last 50 selections (paired *t*-test (9) = 3.1540, *p* < 0.01). In contrast, deck C was chosen significantly more often toward the end of the task compared to the first bin (paired *t*-test (9) = 2.9198, *p* < 0.01; see Figure 1).

### 4.2. *In silico* experiments

#### 4.2.1. Emulating the IGT, adaptative bias.

**Figure 2:**
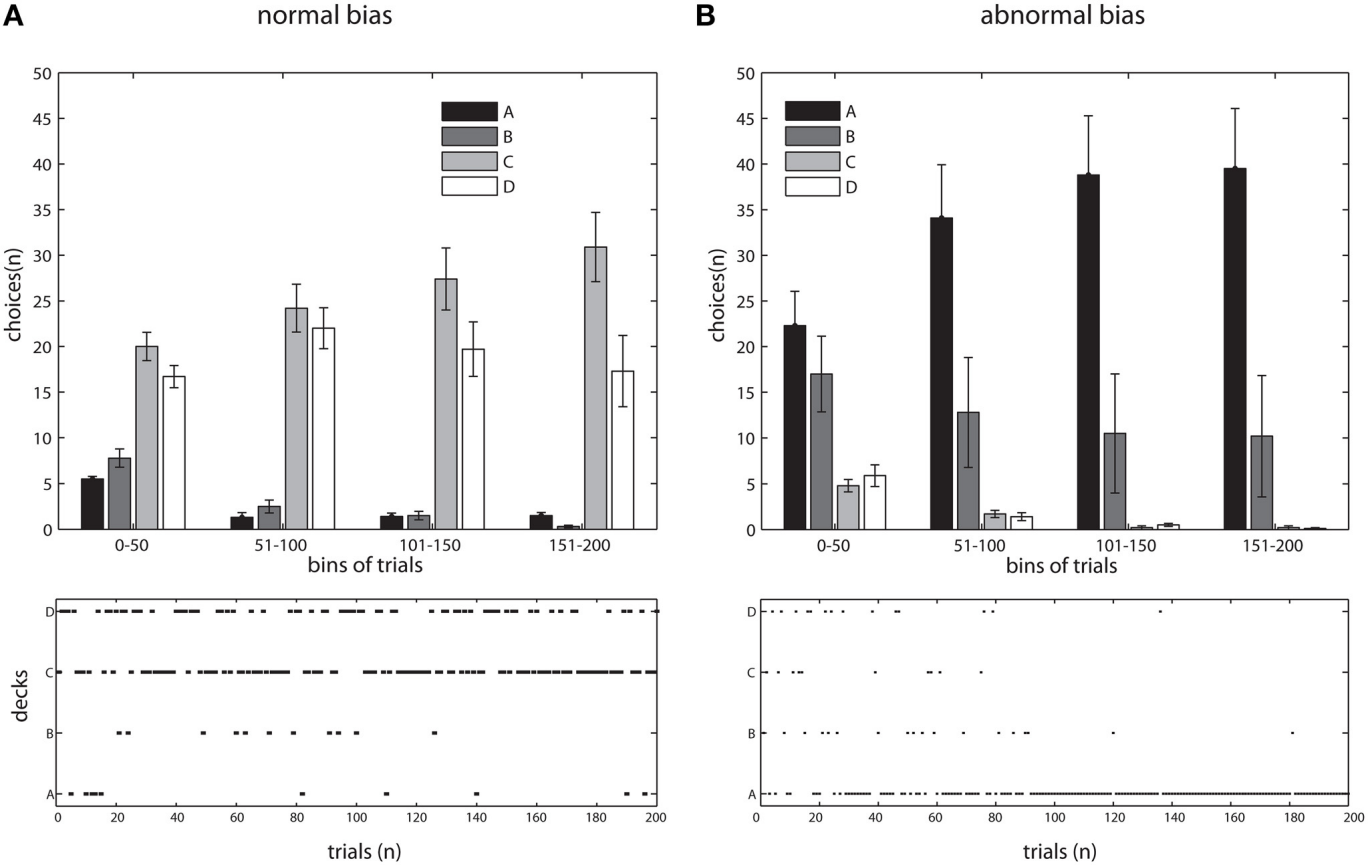
**Simulations of IGT, in adaptive and maladaptive bias**. **(A)** top, Choice's probabilities of 10 instances (participants) across the experiment resembling the adaptive bias deployed by normal subjects in behavioral IGT, pooled in bins of 50 trials. Data as Mean ± S.E.M. bottom, Evolution of choices through the experiment in a typical instance of the simulation, note the normal bias following general trend observed above.

**Figure 3:**
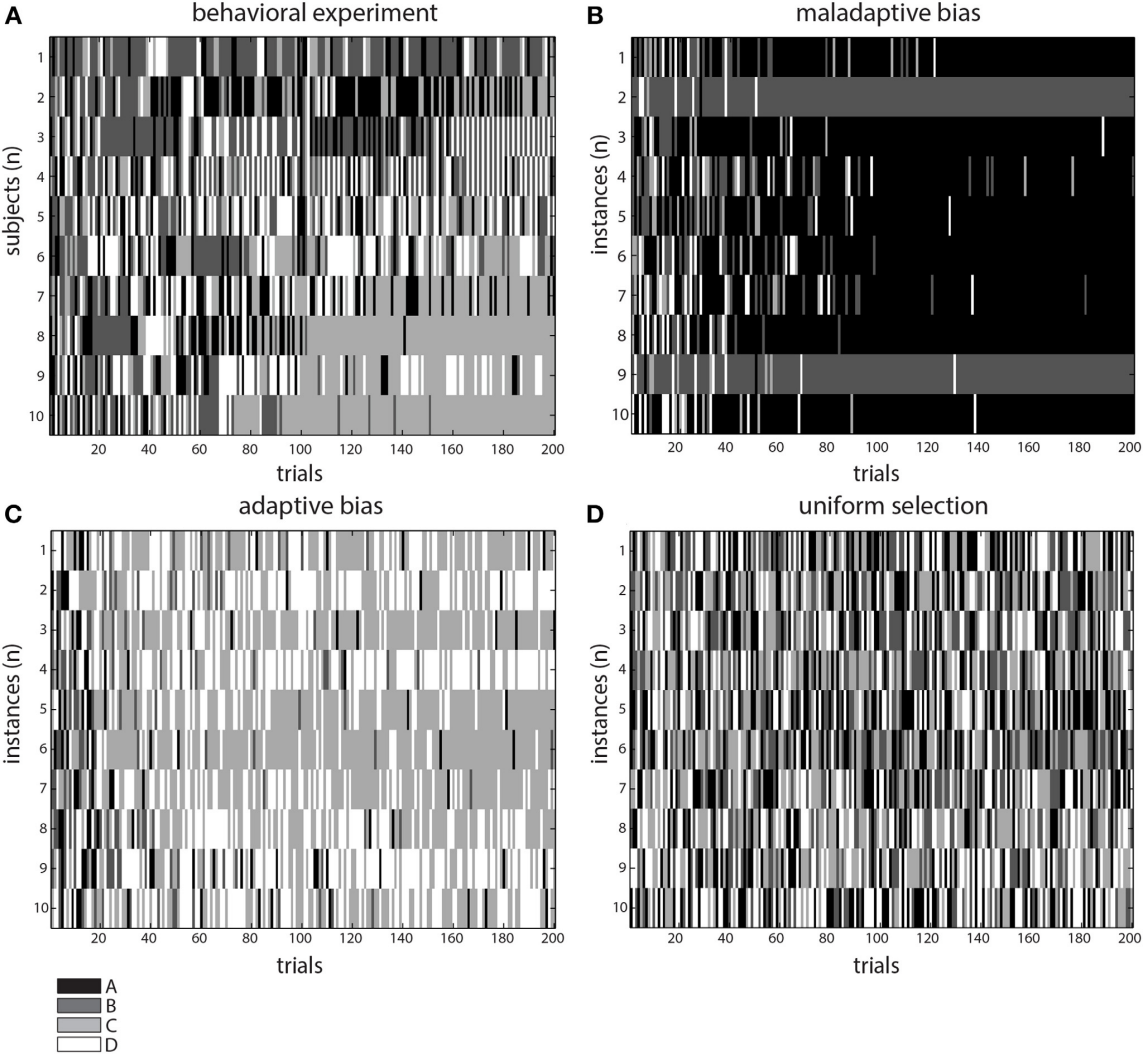
**Comparison of behavioral and simulation results**. Patterns of selection for the behavioral **(A)**, and *in-silico* experiments modeling adaptive **(C)**, maladaptive **(B)** selection biases, and an identical-deck control condition (**D**, see text). Notice the two darker tones **(A,B)** had lower expected outcome, whereas the lighter ones **(C,D)** had higher. Thus, altogether, the gray tones represent a qualitative assessment of the outcome value observed in each experiment.

We started by emulating the IGT administered to healthy participants. The model was used to instantiate an experiment of 200 trials where the distributions of positive and negative feedback for the decks were identical as to the original version of IGT (Bechara et al., 1994). The values for the gain *g*_*k*_ and punishment *l*_*k*_ per ten cards are summarized in Table 1. The resulting selection pattern closely resembled our behavioral data (Lavin et al., 2014) and the historical controls found in the literature (Bechara et al., 1994; Fukui et al., 2005; Buelow and Suhr, 2009; Li et al., 2010). We assessed the data with an Tow-Way ANOVA, with *deck* and *time (bin)* as factors. A main effect of *deck* [*F*_(3, 144)_ = 126.56, *p* < 0.001] as well as a significant *interaction* [*F*_(9, 144)_ = 3.32, *p* < 0.001] were found (see Figures 2A, 3A,C).

#### 4.2.2. Maladaptive bias, IGT in simulated clinical populations.

The IGT was originally administered to vmPFC/OFC patients (Bechara et al., 1994), where researchers observed an inability to update their cognitive representations regarding the underlying distributions of rewards and punishments in the presence of new information. This results in a maladaptive bias toward the inconvenient decks yielding poor performance. In biological terms this impairment was attributed to a dysfunctional interaction between brain areas underlying updating of representations after new contextual information (OFC and Amygdala), differentiation between wins and losses (ACC), and the appropiate use of new information in order to pursue adaptive strategies (vmPFC) (Fukui et al., 2005; Satterthwaite et al., 2007; Li et al., 2010).

In order to contrast this idea, we modified our model to exclude the parameter responsible for updating the reward/punishment probability assigned to each deck. Thus, this modified version assigned deck probabilities in a plain fashion throughout the task and therefore would not deploy an adaptive strategy. We observed a pattern resembling the results from vmPFC/OFC patients, with a greater occurrence of deck choices yielding lower net outcome (decks A and B, see Figures 2B, 3B). We found a main effect of *deck* [*F*_(3, 144)_ = 52.29, *p* < 0.0001], with deck A concentrating the majority of the choices. Interestingly, in this case we did not find an interaction [*F*_(9, 144)_ = 1.64, *p* = 0.1079], a fact that would suggest that the model was not *learning* from the information collected during the experiment.

Finally, we considered an hypothetical case where the model was provided with four identical decks as inputs. In this a case, the intuitive prediction was that the system could not decide on any particular deck and would proceed in selecting the decks randomly, as if it were the first trial. This pattern was indeed observed (see Figure 3D).

## 5. Discussion

In this work we tested a model that emulates people's learning-by-experience processes. We compared the model's performance on the IGT to the behavior of a normal sample of subjects completing this task. We additionally ran two alternative simulations in which we investigated a maladaptive-biased setting that aimed to simulate impaired performances on the IGT, similar to that reported in the clinical literature, and a control condition to explore the interaction between the formal model and its environment.

The behavioral results show that subjects learned from the task because they progressively chose from more advantageous decks as the task progressed. As seen in Figure 1, subjects started choosing decks with higher winnings (mainly deck B), but with experience (between trial 101 and 150) made better decisions by selecting more often from deck C. A similar pattern of exploration is reported in the original IGT research and its replications (Bechara et al., 1994; Crone et al., 2004), finding that subjects initially chose from the deck with higher winnings and progressively switch to the decks that yielded lower rewards but also lower punishments. The cognitive mechanisms underlying the adaptive strategy that subjects develop in the short time during this complex task are associated with emotional responses that signal feedback for guiding future decisions (Bechara et al., 1994, 1996; Martinez-Selva et al., 2006). In this way, the basic cognitive process behind learning under uncertainty is associated with the ways in which past experiences are encoded and later determine the future course of actions. We explored this basic computational mechanism with a formal model based upon such process.

By simulating human interaction during the IGT using a model that integrates this attention to the basic relationship between past and future decisions into a novel equation, we have discretized a stochastic differential equation that takes into account the four possible discrete choices of the IGT at each time step. The equation is based on a Langevin-type of dynamics (Gardiner, 2010). The model allows for the calculation of a new decision based upon the updated probability of each deck in each subsequent trial. Notice how the model takes into account the basic process present in the experiment, without adding any free or dynamic parameter. At the beginning of the interaction the model visits each option in an exploratory way. But as the model interacts with the task, the feedback becomes critical for updating the probabilistic weights of each deck for the next selection.

In order to observe the relevance of feedback information in the model's choices, we ran a control simulation using a modified version of the IGT in which the decks had identical distribution of outcomes. In this condition, the four decks offered the same payoffs and, therefore, no learning occurred during the task. The results showed that the model's choices were symmetrically distributed over the four decks, supporting the importance of feedback for the future choices. The simulation using the standard IGT was expected to replicate the learning trend observed in normal subjects, showing an adaptive bias. The results did show that the choices made by the model were highly concentrated toward the advantageous decks, replicating the trend observed in our behavioral experiment and past studies of healthy subjects playing the IGT (Bechara et al., 1994, 1996; Fukui et al., 2005; Buelow and Suhr, 2009). The simulation results provide support for the function of a cognitive system involved in learning under uncertainty linked to the representation of outcomes and action planning derived from such information (Martinez-Selva et al., 2006; Li et al., 2010).

Varied decision-making paradigms have shown the involvement of brain structures that differentiate between wins and losses (ACC), are responsible for differential emotional reaction toward convenient and inconvenient results (Amygdala), update option values (OFC) and use such information in order to adopt adaptive strategies (vmPFC) (Fukui et al., 2005; Satterthwaite et al., 2007; Li et al., 2010). The SMH provides a framework in which both the amygdala and the vmPFC have a critical role in the interaction of learning processes present during decision making under uncertainty and the related emotional responses. This is relevant because the nature of the explanation rests on the physiological responses associated to the amygdala that differentiate between positive and negative results based on feedback information, and on the cognitive function of the vmPFC for using this information as an anticipatory physiological marker for avoiding disadvantageous choices (Martinez-Selva et al., 2006). These two structures are the basis of the model.

Interestingly, however, there are differences about the timing and the exploratory behavior between the subjects and the results of the adaptive bias simulation. The simulations showed that the trend of choices was already present in the first bin and had less exploration than the behavioral experiment. The model learned to differentiate between convenient and inconvenient decks earlier in the game and, unlike the subjects, was never *tempted* by the decks having higher magnitudes of winnings. A possible explanation for this is that the model processed winnings and losses equally. Kahneman and Tversky provided evidence for the differential impact of winnings and losses in the cognitive system (Kahneman and Tversky, 1984), which has been replicate in several experimental contexts (Edwards, 1996) and also in simulation models such as the PVL and EV-PU descripted previously. Furthermore, there is evidence showing specialized brain areas for outcome processing, including striatal activation after winnings and ACC activity after losses, supporting the idea of cognitive differences in the processing of monetary rewards and punishments (Sanfey, 2007; Lavin et al., 2013). The comparison between the model and the behavioral experiment shows that when economic rewards and punishments are processed equally, there is considerable improvement in IGT performance. In the case of our model we could in the future modify this basic feedback processing with a parameter for the weight of winning and losses and for the shape of the utility function (as in the PVL model), and also modify the parameter that determines the rate of exploration in order to simulate a more accurate exploratory behavior of human decision making under uncertainty. The IGT is a learning task where subjects must encode option values during their interaction with the task. Given that the magnitudes of the winnings (and punishments) are greater in disadvantageous decks, the temptation to earn winnings has a strong influence in the first deck selections that subjects perform. In fact, impaired performances in the IGT are associated with the undifferentiated anticipatory physiological responses between convenient and inconvenient decks (Bechara et al., 1994, 1996). Crone and colleagues showed that normal subjects switched responses more often following punishments than following rewards (Crone et al., 2004). Our results are consistent with these findings because subjects started choosing deck B, which has larger reward magnitude and lower punishment probability, and then progressively switched to deck C. This pattern of choices is similar to the one observed in the PVL model simulation (Steingroever et al., 2013), which supports the idea that the faster learning by the model is related to the way in which the model evaluates outcomes, which is rather simpler to the way humans encode winnings and losses.

The maladaptive-bias simulation aimed to determine whether the model could replicate the behavior observed in clinical populations by modifying the processing of punishments in the IGT. In this condition, the model was programed to learn based upon the positive but not negative feedback, thus it had an inaccurate processing of the feedback given by the choices. Results given by this simulation model revealed persistent choices of disadvantageous decks, similar to behavior by vmPFC/OFC patients (Bechara et al., 1994; Anderson et al., 1999; Bechara and Damasio, 2002; Bechara, 2004). The explanation for the impaired IGT performance in these patients is related to the diminished use of emotional responses that differentiate between positive and negative feedback for conducting future behavior (Bechara et al., 1994, 1996; Crone et al., 2004). Control subjects completing the IGT and good performers of similar gambling tasks show electrodermal response prior to selecting a card from a disadvantageous deck in the IGT (Carter and Smith Pasqualini, 2004; Crone et al., 2004). However, vmPFC/OFC patients do not display this somatic feedback, and instead guide their decisions based solely upon the positive feedback information (Bechara et al., 1994; Bechara and Damasio, 2002). The simple modification of the model biases the decisions in the same way as in such clinical population. Since negative feedback is processed as constant, the model is *tempted* by the winnings, which are higher in the inconvenient decks.

Supporting this interpretation, the persistent behavior of choosing the disadvantageous decks in vmPFC/OFC patients may also indicate impairment in inhibitory control of behavior and response inhibition (Rescorla, 1997). Commonly, a given behavior is repeated when it is rewarded and can change once it is punished. In the IGT, participants must learn over time to inhibit the temptation of choosing decks with higher rewards given the higher punishments of such options. In patients with vmPFC/OFC damage this inhibition may be impaired, and their responses are influenced by immediate feedback rather than longer-term benefits (Rescorla, 1997). Considering our model, if an agent updates the options values without considering losses, then it will persist in a behavior driven by attending to immediate rewards. Since vmPFC has been associated with the integration of emotional and cognitive information during decision-making (Bechara et al., 1994) as well as in controlling impulsive and automatic behavior (Bechara and Van Der Linden, 2005; Buelow and Suhr, 2009), the emotional and cognitive evaluation of future outcomes can affect impulse control. This is consistent with evidence showing poor performances in psychiatric populations having symptoms of impulsivity and anxiety, such as individuals with substance use disorders, pathological gambling, and psychopathy (Buelow and Suhr, 2009). Moreover, damage to the vmPFC could cause working memory problems that may affect IGT performances (Brand et al., 2007), due to learning problems that facilitate persistent maladaptive behavior (Fellows and Farah, 2003; Fellows, 2004).

This study aimed to test whether the cognitive basis of decision-making under uncertainty could be expressed through the stochastic dynamics presented in our model. Results show that the basic equation accounts for part of the computational process involved in such decision-making scenarios, and that simple modification of the feedback processing could provide more realistic emulation of human learning under uncertainty. The research also provided indirect support for the basis of impaired decision making in the IGT, emulating pathological behavior in this task through a simple modification of the equation. Future modeling might explore whether a simple equation that attends to the differential processing of winnings and losses could add more ecological validity to the simulation.

Our findings contribute to a better understanding of the complex constellation of cognitive processes involved in adaptive decision making, implemented by an equally complex circuitry of cortical and subcortical brain areas. We highlighted a core component of such a network, specifically the vmPFC-Amigdala axis, which, as supported by the proposed model, can govern decision making dynamics and critically contribute to behavioral adaptation within unknown environments.

### Conflict of interest statement

The authors declare that the research was conducted in the absence of any commercial or financial relationships that could be construed as a potential conflict of interest.
